# The Reliability of Remote Patient-Reported Outcome Measures via Mobile Apps to Replace Outpatient Visits After Rotator Cuff Repair Surgery: Repetitive Test-Retest Comparison Study for 1-Year Follow-up

**DOI:** 10.2196/20989

**Published:** 2021-03-01

**Authors:** Taek Ho Hong, Myung Ku Kim, Dong Jin Ryu, Jun Sung Park, Gi Cheol Bae, Yoon Sang Jeon

**Affiliations:** 1 Department of Orthopedic Surgery Inha University Hospital Incheon Republic of Korea

**Keywords:** patient-reported outcome measures (PROMs), location, remote PROMs using mobile application, smartphone, mobile phone, follow-up loss

## Abstract

**Background:**

With the development of health care–related mobile apps, attempts have been made to implement remote patient-reported outcome measures (PROMs). In order for remote PROMs to be widely used by mobile apps, the results should not be different depending on the location; that is, remote PROM results performed in locations other than hospitals should be able to obtain reliable results equivalent to those performed in hospitals, and this is very important. However, to our knowledge, there are no studies that have assessed the reliability of PROMs using mobile apps according to the location by comparing the results performed remotely from the hospital and performed at the outpatient visits.

**Objective:**

The purpose of this study was to evaluate the reliability of remote PROMs using mobile apps compared to PROMs performed during outpatient follow-up visits after arthroscopic shoulder surgery.

**Methods:**

A total of 174 patients who underwent arthroscopic rotator cuff repair completed questionnaires 2 days before visiting the clinic for the 1-, 2-, 3-, 6-, and 12-month follow-ups (test A). The patients completed the questionnaires at the clinic (test B) using the same mobile app and device for the 1-, 2-, 3-, 6-, and 12-month follow-ups. Test-retest comparisons were performed to analyze the differences and reliability of the PROMs according to the period.

**Results:**

Comparisons of tests A and B showed statistically significant differences at 1, 2, and 3 months (all *P*s<.05 except for the ASES function scale at 3-months) but not 6 or 12 months after surgery (all *P*s>.05). The intraclass correlation values between the two groups were relatively low at the 1-, 2-, and 3-month follow-ups but were within the reliable range at 6 and 12 months after surgery. The rate of completion of tests A and B using the mobile app was significantly lower in the group older than 70 years than in the other groups for all postoperative periods (*P*<.001).

**Conclusions:**

PROMs using mobile apps with different locations differed soon after surgery but were reliably similar after 6 months. The remote PROMs using mobile apps could be used reliably for the patient more than 6 months after surgery. However, it is to be expected that the use of mobile app–based questionnaires is not as useful in the group older than 70 years as in other age groups.

## Introduction

### Background

With the dramatically increased penetration rates worldwide [1], at 81% in the United States and 95% in South Korea [2], smartphones are becoming increasingly indispensable in everyday life [3]. A variety of mobile apps for information, communication, education, and entertainment purposes have been developed for smartphones [3], including mobile health care systems. Seto et al [4] developed a mobile phone–based telemonitoring program for patients with heart failure following acute decompensation. Denono et al [5] suggested that postoperative mobile apps after ambulatory lumbar discectomy were effective tools for spine surgeons.

With the development of health care–related mobile apps, attempts have been made to implement remote patient-reported outcome measures (PROMs). Skrepnik et al [6] assessed the impact of a novel smartphone app compared with standard follow-up on mobility following treatment with intra-articular injection in patients with knee osteoarthritis. Armstrong et al [7] evaluated the effect of home monitoring via a mobile app on the number of in-person visits following ambulatory surgery. Most studies reported that patients found mobile apps for remote follow-ups to be convenient, safe, and highly satisfactory [4-8]. Reliable remote follow-ups by mobile health care systems have several advantages over face-to-face follow-ups. In general, follow-up durations of at least 12 months to several years are required for reliable clinical study findings after surgery [9,10]. However, maintaining high rates of long-term follow-up is challenging due to poor patient compliance [10,11]. Remote follow-ups using mobile PROMs are also efficient in terms of health care costs compared to outpatient visits [12]. Considering the difficulty in long-term follow-up [10], the reduction in outpatient follow-ups, and the reduced health care costs [12], PROMs using mobile apps performed outside of clinics may be good alternatives. In order for remote PROMs to be widely used by the mobile app, the results should not be different depending on the location; that is, remote PROM results performed in locations other than hospitals should be able to obtain reliable results equivalent to those performed in hospitals, and this is very important. However, to our knowledge, there are no studies that have assessed the reliability of PROMs using mobile apps according to the location by comparing the results performed remotely from the hospital and performed at the outpatient visits.

### Goal of This Study

Therefore, this study evaluated the reliability of remote PROMs using mobile apps compared to the PROMs performed by the same mobile apps during outpatient follow-up visits after arthroscopic shoulder surgery. We also analyzed the tendencies in differences with increasing time after surgery and observed the PROM participation rates of patients according to the follow-up periods with repetitive test-retest studies. We hypothesized that the results of the PROMs would be similar between those measured in outpatient clinic visits and those measured remotely using mobile apps.

## Methods

### Patients and Study Design

**Figure 1:**
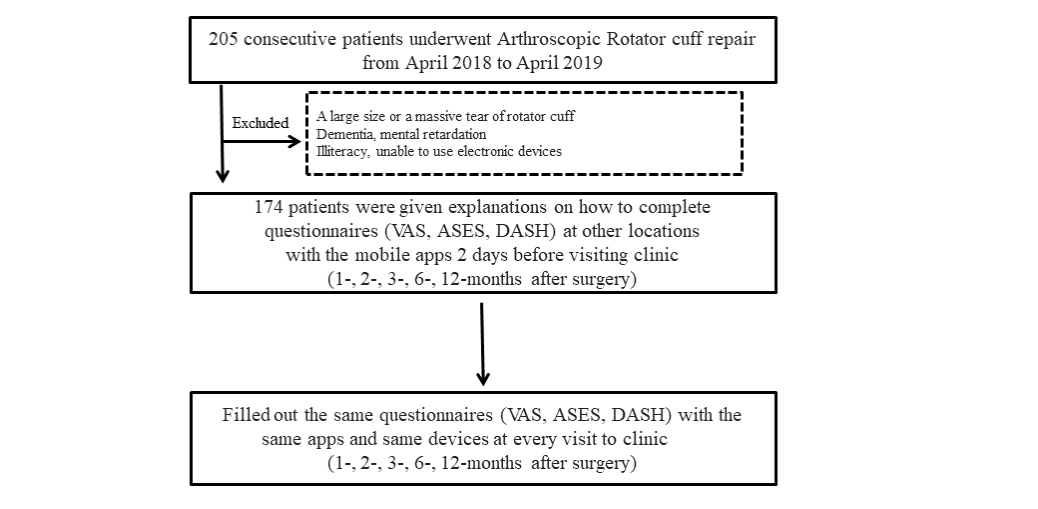
Inclusion and exclusion criteria for the study. ASES: American Shoulder and Elbow Society; DASH: Disabilities of the Arm, Shoulder, and Hand; VAS: visual analog scale.

205 consecutive patients who underwent arthroscopic rotator cuff repair by a single surgeon were initially considered for this study between April 2018 and April 2019. Patients diagnosed with large or massive rotator cuff tears were excluded because of the difference in their rehabilitation schedules. Patients with dementia, mental retardation, illiteracy, or inability to use electronic devices were excluded because of the difficulty in completing questionnaires using electronic equipment. After exclusion, the remaining 174 patients (92 men and 82 women) prospectively conducted the test-retest comparisons, which were performed 5 times each to assess the results after surgery. The patients were instructed to complete questionnaires (visual analog scale [VAS], American Shoulder and Elbow Society [ASES] scale [13], and Disabilities of the Arm, Shoulder, and Hand [DASH] scale [14]) at other locations (test A) 2 days before visiting the clinic for the 1-, 2-, 3-, 6-, and 12-month postoperative follow-ups. Using the same app and electronic devices, namely, their mobile phones, each patient completed the same questionnaires at the clinic (test B) at 1, 2, 3, 6, and 12 months after surgery (Figure 1). The patients received mobile messages linked to an app for an electronic PROM system (Proscore, Incheon, South Korea). All patients who visited our clinic answered the same questionnaires with the mobile app before treatment. The timing of mobile messaging was determined to be 48 hours before the clinic visit based on a previous systematic review that reported test-retest reliability [15]. Of the 174 patients, test A (PROMs completed via the mobile app installed on the mobile phone of each patient 2 days before the clinic visit) was completed by 148 at 1 month, 135 at 2 months, 106 at 3 months, 77 at 6 months, and 59 at 12 months. All 174 patients visited our clinic at 1 month after surgery. However, the rates of outpatient visits with patients completing test B using the same app and electronic devices (the mobile phone of each patient) decreased over time, with 170 visiting at 2 months, 142 at 3 months, 112 at 6 months, and 95 at 12 months after surgery (Figure 1). All patients underwent the same course of rehabilitation. An abduction brace was applied for 4 weeks after surgery. Passive range of motion exercises were allowed from 4 to 8 weeks after surgery. Active range of motion exercises were conducted 8 weeks after surgery. This study, including the subject selection and data collection, was conducted under the approval of the Inha University Hospital Institutional Review Board (IRB INHA 2019-09-024) in accordance with the 1964 Declaration of Helsinki.

### Scale Definitions and Measures

The VAS score is measured ranging from 0 to 10, with scores of 0 and 10 indicating “no pain” and “worst pain imaginable,” respectively. The ASES scale [13] consists of two subscales, namely, pain (1 item) and function (10 items). Each subscale is transformed to scores ranging from 0 to 50, based on patient responses. The sum of the two scales is the total score on the ASES scale, with a score of 100 points indicating perfect conditions of the shoulder. This study analyzed the total ASES scale score as well as the scores for the two subscales. The DASH scale comprises 30 items (21 on daily activities, 5 on symptoms, 3 on participation, and 1 on confidence in ability) [14]. Higher scores indicate worse upper limb function. We used an electronic PROM system (Proscore, Incheon, South Korea) available as an app for electronic devices that measures VAS, ASES scale, and DASH scale scores at locations other than the clinic. In this system, patients touched the answer on the screen instead of marking their responses on original paper questionnaires using a writing instrument. This change from paper-based to electronic-based measures is minor, according to the Food and Drug Administration guidelines [16].

### Statistical Analyses

The data are expressed as means (standard deviations) or medians (ranges). Paired *t* tests (2-tailed) were used to evaluate differences between the answers for tests A and B; more specifically, the average score with standard deviations of the scale’s scores was calculated and analyzed using paired *t* tests. We also calculated the average absolute value of the differences between tests A and B. Intraclass correlation coefficients (ICCs) were calculated to estimate reproducibility and reliability between tests A and B. Statistical significance was indicated by *P*<.05. All statistical analyses were performed using IBM SPSS Statistics for Windows, version 19.0 (IBM Corp, Armonk, NY).

## Results

The demographics of patients undergoing rotator cuff surgery are summarized in Table 1.

**Figure 2:**
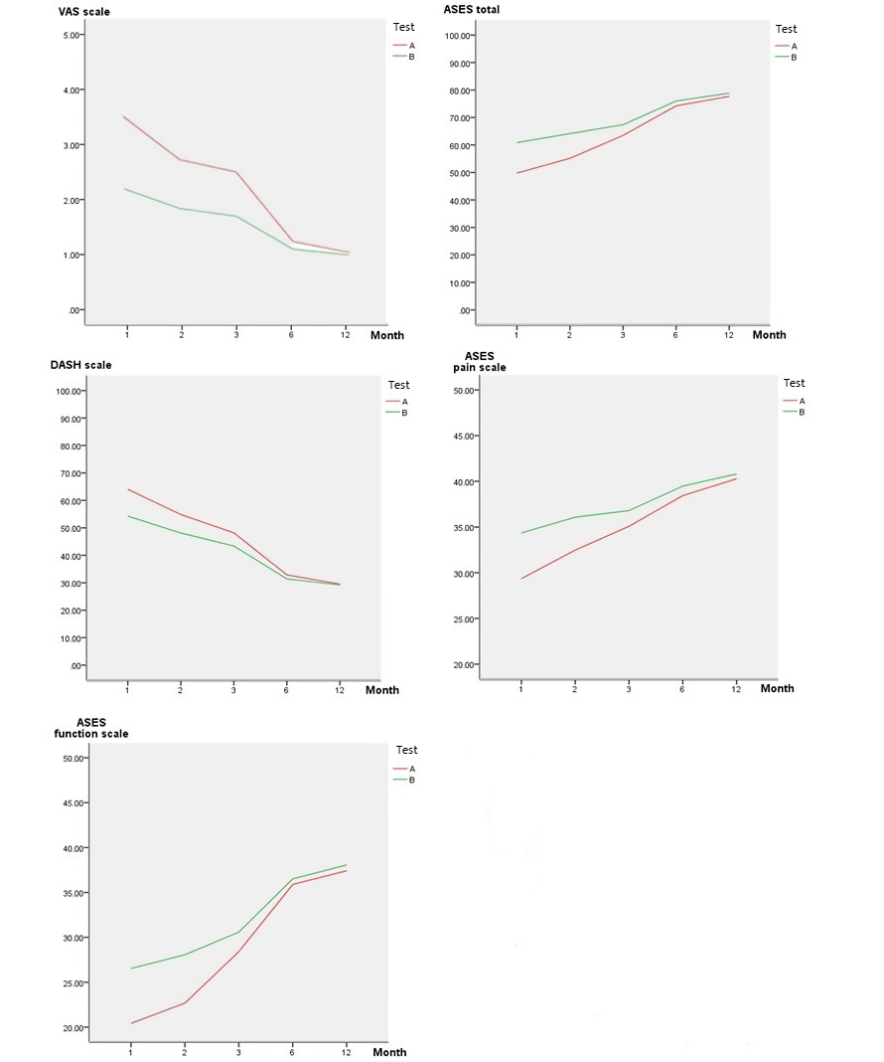
Mean (standard deviation) for each scale used in this study. Data presented for 5 tests by postoperative duration (N=174). ASES: American Shoulder and Elbow Society; DASH: Disabilities of the Arm, Shoulder, and Hand; VAS: visual analog scale.

The average scores and absolute values of the differences between tests A and B are shown in Table 2 and Figure 2 for the 1-, 2-, 3-, 6-, and 12-month postoperative results. At 1, 2, and 3 months after surgery, test B showed significantly better outcomes compared to those of test A (*P*s<.05), except for the ASES function subscale (*P*=.06 at 3 months). All parameters did not show statistically significant differences (all *P*s>.05) between tests A and B at 6 and 12 months after surgery. The average absolute differences in VAS, ASES total, and DASH scores between tests A and B were 1.68, 14.72 and 11.28 points at 1 month after surgery, respectively. In most of the scales, the differences in the average and absolute differences gradually decreased with time after surgery. At 12 months after surgery, the average absolute value differences in VAS, ASES total, and DASH scores between tests A and B were greatly reduced (0.32, 5.48, and 4.46 points, respectively).

**Table 1:** Baseline demographic and clinical characteristics (N=174).

Characteristic		Value
Age (years), mean (SD)		59.38 (10.9)
Gender, female, n (%)		82 (47.1)
**Side, n (%)**		
	Right	97 (55.7)
	Left	77 (44.3)
Symptom duration (months), mean (SD)		11.18 (13.74)
**Tear size, n (%)**		
	Small	96 (55.2)
	Medium	78 (44.8)

**Table 2:** Mean (standard deviation) for each scale by 1-, 2-, 3-, 6-, and 12-month postoperative data analyzed by paired *t* test or Wilcoxon signed rank test (N=174).

POD^a^ and scale		Test A, mean (SD)	Test B, mean (SD)	Differences	*P* value	Absolute differences^b^
**POD 1 month**						
	VAS^c^ score	3.23 (1.69)	1.96 (1.16)	1.27	<.001	1.68 (1.23)
	ASES^d^ total	50.02 (11.78)	60.56 (14.85)	−10.54	<.001	14.72 (10.07)
	ASES pain	29.42 (8.85)	34.32 (11.73)	−4.90	<.001	11.28 (7.53)
	ASES function	20.60 (6.40)	26.24 (7.51)	−5.64	<.001	7.53 (6.26)
	DASH^e^	64.01 (10.57)	53.82 (11.92)	10.19	<.001	12.94 (9.35)
**POD 2 months**						
	VAS score	2.44 (1.62)	1.60 (1.17)	0.84	<.001	1.25 (1.04)
	ASES total	54.88 (15.93)	63.68 (12.89)	−8.80	<.001	13.85 (9.41)
	ASES pain	32.55 (12.85)	35.96 (9.35)	−3.41	.003	10.88 (8.28)
	ASES function	22.33 (7.62)	27.72 (8.52)	−5.39	<.001	8.40 (6.46)
	DASH	54.26 (9.82)	48.34 (11.81)	5.92	<.001	9.49 (6.93)
**POD 3 months**						
	VAS score	2.24 (1.64)	1.54 (1.05)	0.70	.03	1.28 (0.95)
	ASES total	62.73 (12.05)	67.15 (11.62)	−4.42	.01	11.43 (9.10)
	ASES pain	34.57 (9.93)	36.88 (8.00)	−2.31	.02	7.87 (6.36)
	ASES function	28.16 (6.82)	30.27 (7.37)	−2.11	.06	6.60 (5.35)
	DASH	48.10 (9.26)	43.36 (12.32)	4.74	<.001	8.86 (7.06)
**POD 6 months**						
	VAS score	1.06 (0.54)	0.88 (0.70)	0.18	.13	0.46 (0.59)
	ASES total	74.28 (12.15)	77.19 (13.20)	−2.91	.09	8.78 (7.09)
	ASES pain	37.07 (8.86)	39.35 (7.83)	−2.28	.21	5.90 (5.94)
	ASES function	37.20 (7.82)	37.84 (9.69)	−0.64	.30	5.26 (4.82)
	DASH	36.64 (10.95)	33.10 (9.54)	3.54	.48	7.73 (4.94)
**POD 12 months**						
	VAS score	0.83 (0.56)	0.77 (0.58)	0.06	.49	0.32 (0.47)
	ASES total	78.78 (9.07)	79.96 (10.94)	−1.18	.17	5.48 (3.76)
	ASES pain	41.69 (6.53)	42.45 (5.67)	−0.76	.24	3.81 (3.75)
	ASES function	37.08 (7.32)	37.51 (8.87)	−0.43	.32	3.47 (2.80)
	DASH	31.34 (8.81)	30.29 (7.66)	1.05	.19	4.46 (3.74)

^a^POD: postoperative duration.

^b^Absolute differences are calculated by taking the greater value minus the smaller one between tests A and B.

^c^VAS: visual analog scale.

^d^ASES: American Shoulder and Elbow Society Shoulder Index.

^e^DASH: Disabilities of the Arm, Shoulder, and Hand score.

To estimate the reproducibility and reliability between tests A and B, ICC values were calculated for each scale and subscale (Table 3). The VAS scale and ASES pain subscale showed relatively low ICC values compared to those of the other scales. The lowest ICC value for the VAS scale was observed at 1 month after surgery (0.51, moderate reliability). The low ICC values for the ASES pain subscale were observed at 1, 2, and 3 months after surgery (0.47, 0.46, and 0.47, respectively; poor reliability). Moderate ICC values were observed for the ASES function subscale at 1, 2, and 3 months after surgery (0.50, 0.53, and 0.67, respectively). At 6 months after surgery, all parameters showed good ICC values (0.77 for VAS, 0.83 for DASH scale, 0.80 for ASES function subscale, 0.78 for ASES pain subscale, and 0.78 for ASES total scale). Regarding the DASH scale, a good ICC value was observed at 6 months after surgery (0.83). The highest ICC values for all parameters were observed at 12 months after surgery. VAS score, ASES pain subscale, and DASH scale showed good ICC values at 12 months after surgery (0.81, 0.76, and 0.87, respectively). The ASES function scale and the ASES total scale showed excellent ICC values at 12 months after surgery (0.91 and 0.90, respectively).

**Table 3:** Intraclass correlation coefficient values for each scale (N=174).

Scale/subscale		Postoperative duration (months)				
		1	2	3	6	12
VAS^a^ pain		0.51	0.67	0.62	0.77	0.81
**ASES^b^**						
	Pain	0.47	0.46	0.47	0.78	0.76
	Function	0.54	0.65	0.67	0.8	0.91
	Total	0.5	0.53	0.58	0.78	0.9
DASH^c^ total		0.57	0.72	0.71	0.83	0.87

^a^VAS: visual analog scale.

^b^ASES: American Shoulder and Elbow Society Shoulder Index.

^c^DASH: Disabilities of the Arm, Shoulder and Hand score.

The rates of outpatient visits and completions of tests A and B according to the period for each age group are shown in Table 4. At 1 month, all 174 patients visited our clinic. However, with time after surgery, the number of outpatient visits gradually decreased. No significant differences in the numbers of outpatient visits were observed in terms of age (*P*=.60, .54, .91, and .70 for 2, 3, 6, and 12 months after surgery, respectively). The rate of completion of tests A and B using the mobile app was significantly lower in the group older than 70 years than in the other groups for all postoperative periods (*P*<.001).

**Table 4:** Visit rate and test-retest response rate by age at each postoperative period.

Rates by postoperative period (months)		Age (years), n (%)					*P* value
		<50 (n=33)	50-59 (n=51)	60-69 (n=52)	≥70 (n=38)	Total (N=174)	
**Outpatient visits (visit rate)**							
	1	33 (100)	51 (100)	52 (100)	38 (100)	174 (100)	—
	2	32 (97.0)	51 (100)	50 (96.2)	37 (97.4)	170 (97.7)	.60
	3	28 (84.8)	42 (82.4)	44 (84.6)	28 (73.7)	142 (81.6)	.54
	6	20 (60.6)	32 (62.7)	34 (65.4)	26 (68.4)	112 (64.4)	.91
	12	15 (45.5)	29 (56.9)	30 (57.7)	21 (55.3)	95 (54.6)	.70
**Test-retest responses (response/visit rate)**							
	1	32 (97.0)	48 (94.1)	47 (90.4)	21 (55.3)	148 (85.1)	<.001
	2	30 (93.7)	45 (88.2)	43 (86.0)	17 (45.9)	135 (79.4)	<.001
	3	25 (89.3)	37 (88.1)	36 (81.8)	8 (28.6)	106 (74.6)	<.001
	6	19 (95.0)	26 (81.2)	25 (73.5)	7 (26.9)	77 (68.7)	<.001
	12	13 (86.7)	22 (75.9)	19 (63.3)	5 (23.8)	59 (62.1)	<.001

In this study, 36 of 174 patients (20.7%) completed all follow-up visits (1, 2, 3, 6, and 12 months after surgery) and also completed tests A and B (completely implemented group). We also performed comparisons between tests A and B in this group to determine the average difference for each scale (Table 5). The ICC values between tests A and B in the completely implemented group (n=36) were similar to those for all 174 patients (Table 6). At 1, 2, and 3 months after surgery, test B showed significantly better outcomes than those of test A (*P*s<.05), except for the ASES pain subscale and DASH scale. No parameter differed significantly between tests A and B at 6 and 12 months after surgery. The average absolute value of the differences for the VAS, ASES total, and DASH scores between tests A and B were 1.50, 15.97, and 10.28 points, respectively, at 1 month after surgery. In most of the scales, the average and the absolute differences gradually decreased with time after surgery. All parameters showed poor or moderate ICCs at 1, 2, and 3 months after surgery but showed moderate or good values at 6 months and peaked at 12 months after surgery for all parameters. The VAS score, ASES pain subscale, and ASES total scale showed good ICCs at 12 months after surgery (0.80, 0.82, and 0.88 respectively), while the ASES function scale and the DASH scale showed excellent ICCs at 12 months after surgery (0.92 and 0.90, respectively).

**Table 5:** Mean (standard deviation) for each scale by 1-, 2-, 3-, 6-, and 12-month postoperative data analyzed by paired *t* test or Wilcoxon signed rank test in the completely implemented group (N=36).

POD^a^ and scale		Test A, mean (SD)	Test B, mean SD)	Differences	*P* value	Absolute differences^b^
**POD 1 month**						
	VAS^c^ score	3.05 (1.58)	1.88 (1.06)	1.17	.009	1.50 (1.02)
	ASES^d^ total	51.24 (10.84)	63.19 (13.11)	−11.95	<.001	15.97 (9.92)
	ASES pain	30.27 (9.01)	35.97 (10.33)	−5.70	.02	10.28 (7.35)
	ASES function	20.96 (7.18)	27.21 (7.23)	−6.25	<.001	8.10 (5.97)
	DASH^e^	64.75 (12.68)	55.48 (11.39)	9.27	<.001	13.41 (8.86)
**POD 2 months**						
	VAS score	2.41 (1.61)	1.55 (1.22)	0.86	.004	1.47 (1.05)
	ASES total	56.94 (12.20)	66.75 (9.08)	−9.81	<.001	14.81 (9.99)
	ASES pain	33.33 (13.09)	37.78 (8.49)	−3.45	.03	10.01 (7.36)
	ASES function	23.60 (6.75)	28.97 (7.42)	−5.37	<.001	8.14 (5.26)
	DASH	54.20 (9.82)	47.19 (10.20)	7.01	<.001	9.54 (9.15)
**POD 3 months**						
	VAS score	2.24 (1.64)	1.54 (1.05	0.70	.01	1.23 (1.09)
	ASES total	62.51 (12.07)	67.15 (11.62)	−4.64	.02	12.73 (9.82)
	ASES pain	34.57 (9.93)	36.88 (8.00)	−2.31	.07	7.77 (6.80)
	ASES function	27.93 (6.65)	30.26 (7.37)	−2.33	.03	7.54 (5.51)
	DASH	48.10 (9.26)	43.36 (12.32)	4.74	.18	8.85 (6.81)
**POD 6 months**						
	VAS score	1.06 (0.54)	0.88 (0.70)	0.18	.12	0.53 (0.60)
	ASES total	74.28 (12.15)	77.19 (13.20)	−2.91	.18	9.16 (7.17)
	ASES pain	37.07 (8.86)	39.35 (7.83)	−2.28	.09	5.69 (6.67)
	ASES function	37.20 (7.82)	37.84 (9.69)	−0.64	.73	5.51 (5.09)
	DASH	36.64 (10.95)	33.10 (9.54)	3.54	.28	6.49 (4.36)
**POD 12 months**						
	VAS score	0.83 (0.56)	0.77 (0.58)	0.06	.17	0.36 (0.48)
	ASES total	78.78 (9.07)	79.96 (10.94)	−1.18	.16	4.67 (3.39)
	ASES pain	41.69 (6.53)	42.45 (5.67)	−0.76	.15	3.61 (3.50)
	ASES function	37.08 (7.32)	37.51 (8.87)	−0.43	.56	3.38 (3.07)
	DASH	31.34 (8.81)	30.29 (7.66)	1.05	.41	3.61 (2.52)

^a^POD: postoperative duration.

^b^Absolute differences are calculated by taking the greater value minus the smaller one, between tests A and B.

^c^VAS: visual analog scale.

^d^ASES: American Shoulder and Elbow Society Shoulder Index.

^e^DASH: Disabilities of the Arm, Shoulder, and Hand score.

**Table 6:** Intraclass correlation coefficient values for the completely implemented group (N=36).

Scale/subscale		Postoperative duration (months)				
		1	2	3	6	12
VAS^a^ pain		0.45	0.54	0.53	0.76	0.80
**ASES^b^**						
	Pain	0.53	0.56	0.54	0.78	0.82
	Function	0.56	0.5	0.58	0.81	0.92
	Total	0.56	0.52	0.57	0.81	0.88
DASH^c^ total		0.56	0.60	0.53	0.84	0.90

^a^VAS: visual analog scale.

^b^ASES: American Shoulder and Elbow Society Shoulder Index.

^c^DASH: Disabilities of the Arm, Shoulder and Hand score.

## Discussion

The results of this study revealed that the PROMs varied depending on the location for the initial 1-, 2-, and 3-month follow-ups after arthroscopic shoulder surgery. However, at 6 months or more after surgery, the PROMs using the mobile apps showed similar results regardless of location. The ICC analysis also showed a tendency toward relatively low values for 1, 2, and 3 months postoperatively according to the PROM location, while high values were recorded at the 6- and 12-month follow-ups. These findings indicated that PROMs performed using mobile apps at 6 months after surgery were adequately reliable and reproducible regardless of location. Therefore, the use of remote PROMs via mobile apps may be more valuable for follow-ups at 6 months or more after surgery, when the rate of follow-up loss is increased.

Most scales showed different outcomes for test B compared to those for test A at the initial 1, 2, and 3 months postsurgery. However, at 6 and 12 months after surgery, none of the scales differed significantly between tests A and B. The absolute values of the differences were also greatly reduced with time, and the reliability as assessed by ICC was adequately high after 6 months. These outcomes are consistent with those of previous studies on the test-retest reliability of PROMs. Chahal et al [17] reported good reliability of PROM for knee joint–specific questionnaires in a test-retest study conducted 6 months after multiligament knee injury. Bramming et al [18] reported that a PROM (forgotten joint score-12) showed high relative reliability in a test-retest study conducted at 6 months after hip arthroscopic surgery. The differences in follow-ups performed in the first 3 months postsurgery might be due to variability in patient conditions during the acute phase following surgery. Additionally, the differences may have decreased over time due to patients getting used to the test items by repeatedly performing PROMs. The absolute values of the differences between the two tests were also noteworthy, given that the purpose of this study was to measure the difference between outpatient and remote mobile apps. The absolute values of the differences for each scale were relatively high at the 1-, 2-, and 3-month follow-ups. However, at the 6- and 12-month follow-ups, all parameters showed reduced absolute differences. These results also reinforce the reliability of the remote PROMs compared to outpatient PROMs for long-term follow-ups.

Clinical studies on patient outcomes after surgery generally require at least 12 months to several years of follow-up for recognition as reliable clinical studies [9,10]. To avoid biases in clinical studies using PROMs performed at the clinic, it is important to minimize loss to follow-up to the hospital [19,20]. However, maintaining high rates of long-term follow-up is challenging due to poor patient compliance [10]. Cronin et al [21] showed that 40% of patients with orthopedic trauma did not complete 90 days of follow-up. Zelle et al [19] also reported that patients with undifferentiated orthopedic trauma showed high rates (>70%) of noncompliance in the initial 6 months postsurgery. Considering that patients' compliance with outpatient follow-up decreases over time after surgery [21], the reliability of PROMs via mobile apps regardless of the location for long-term follow-up after surgery is meaningful as these PROMs may be an option to assess patient condition without a need to travel to the hospital.

Even in terms of the cost benefits and efficient follow-ups for patients [12], the reliability of remote PROMs is also important. Higgins et al [22] compared a conventional in-person visit follow-up group (conventional group) to a non–face-to-face follow-up group using a mobile app (mobile app group) for 6 weeks after anterior cruciate ligament reconstruction. The mobile app group had 0.36 clinic visits during the study period, compared to 2.44 visits in the conventional group. The mobile app group spent Can $211 (US $166.16) less over 6 weeks than the other group. Thus, in terms of cost burden, remote PROMs may also have advantages over outpatient visits if the assessments are reliable.

Due to the recent infectious disease epidemics of COVID-19 [11], it is difficult to expect patients to comply with outpatient follow-ups in the absence of an emergency [23]. Remote PROMs are particularly valuable [11] as medical staff and national health care system resources are focused on a particular infectious disease [24-26]. Recent guidelines from the Journal of Bone and Joint Surgery [27] recommend the assessment of all planned elective or nonemergency surgical procedures and clinical visits to determine whether they can be postponed or canceled. If remote PROMs are reliable, they can be effective and highly utilized for reducing patient visits [11] and allow efficient distribution of the national health care system capacity when infectious disease outbreaks occur.

This study used an electronic PROM system (Proscore, Incheon, South Korea) available for mobile phones. The correlation between electronic measuring systems and conventional paper-and-pencil methods is reportedly reliable [28]. The compliance of patients for completing scoring tools using electronic systems is generally better than that for paper-and-pencil methods because it is more convenient and quicker [29]. However, older patients may not prefer performing PROMs with electronic devices because of less exposure to and familiarity with electronic devices compared to younger patients [29]. In this study, the rate of outpatient visits did not differ significantly by age; however, the rates of test-retest completion for both PROMs at outpatient visits and remote PROMs using mobile apps were statistically significantly lower in patients older than 70 years than those in other groups for all postoperative periods (*P*<.001). Instructions for the use of smartphone devices and apps must be provided to the elderly in order to use PROMs via mobile apps at locations other than hospitals.

Test-retest assessments to evaluate the reliability of tools are generally conducted once for comparisons. However, this study conducted test-retest comparisons 5 times each to determine the tendencies with increasing time after surgery, which is a strength of this study. The limitations of this study were its inclusion of only patients who underwent arthroscopic rotator cuff repair. However, this could also be considered a strength as confounding variables due to many disease entities are reduced. Several diseases and treatment options for the shoulder joint, including intra-articular injection for frozen shoulder, reverse total shoulder arthroplasty for rotator cuff arthropathy, and other disease categories, might be candidates for further study.

In conclusion, PROMs performed using mobile apps in different locations showed varied results soon after surgery but were similar after 6 months, with reliable ICC values. The remote PROMs using mobile apps could be used reliably for the patient more than 6 months after surgery. However, it is to be expected that the use of mobile app–based questionnaires is not as useful in the group older than 70 years as in other age groups.

## Abbreviations

ASES: American Shoulder and Elbow Society
DASH: Disabilities of the Arm, Shoulder, and Hand
ICC: intraclass correlation coefficient
PROMs: patient-reported outcome measures
VAS: visual analog scale
